# Gastric Endocrine Cell Carcinoma Coexistent with Adenocarcinoma

**DOI:** 10.1155/2013/502451

**Published:** 2013-12-25

**Authors:** Nobuhiro Takeuchi, Nomura Yusuke, Tetsuo Maeda, Kazuyoshi Naba

**Affiliations:** ^1^Department of Gastroenterology, Kawasaki Hospital, Kobe, Hyogo 652-0042, Japan; ^2^Department of Laboratory Medicine, Kawasaki Hospital, Kobe, Hyogo 652-0042, Japan

## Abstract

A 69-year-old female presented to our institution with epigastralgia and abdominal distension. Upper gastrointestinal series revealed a 5 cm ulcerative lesion with irregular margins and elevated distinct borders from the angle to the pyloric ring. Gastroendoscopy revealed a Borrmann type 2 tumor. Several biopsied specimens revealed proliferation of small and heterogeneous cancer cells with rich chromatin and fibrous septum with rich vessels at connective tissues, which was confirmed as gastric endocrine cell carcinoma (ECC) on immunostaining with chromogranin and synaptophysin. Furthermore, other specimens revealed atypical cells forming glandular structures, which were confirmed as well-differentiated tubular adenocarcinomas. Distal gastrectomy with D2 lymph node dissection and Billroth I reconstruction was performed. Pathological examination of the gross specimen revealed that adenocarcinoma comprised <10% of all cancer cells. Close analysis of ECC revealed a mixture of small and large cells. According to the WHO 2010 classification of gastrointestinal neuroendocrine tumors, this gastric tumor was diagnosed as neuroendocrine carcinoma. The patient was administered adjuvant chemotherapy with cisplatin and etoposide. One year following surgery, follow-up abdominal CT revealed multiple liver metastases. The patient received the best supportive care but eventually died 18 months after surgery. Here we present this case of gastric ECC coexistent with adenocarcinoma.

## 1. Introduction

Gastric endocrine cell carcinoma (ECC), characterized by endocrine differentiation and aggressive biological behavior, is occasionally accompanied by the presence of adenocarcinoma cells. On the basis of the analysis of p53 gene alteration, the hypothesis that was derived proved that adenocarcinoma cells have the potential to develop into gastric ECC [1]. Here we present a case of gastric ECC coexistent with adenocarcinoma as well as p53 gene analyses of ECC and adenocarcinoma.

## 2. Case Presentation

**Figure 1:**
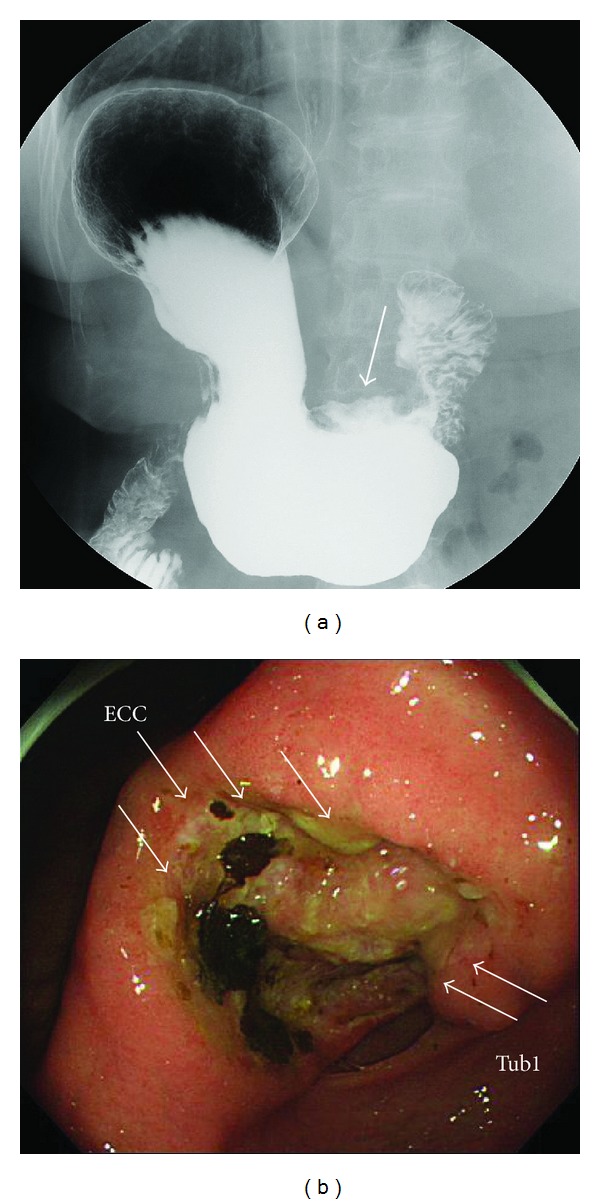
Upper gastrointestinal series revealed a 5 cm ulcerative lesion with irregular margins and elevated distinct borders from the angle to the pyloric ring (a). Gastroendoscopy revealed a Borrmann type 2 tumor, extending from the angle to the antrum of the lesser curvature (b). The tumor comprised two different parts: ulcers with severe invasiveness and smooth protrusion from the endocrine cells and well-differentiated adenocarcinoma cells, as revealed by specimens biopsied under gastroendoscopy.

**Figure 2:**
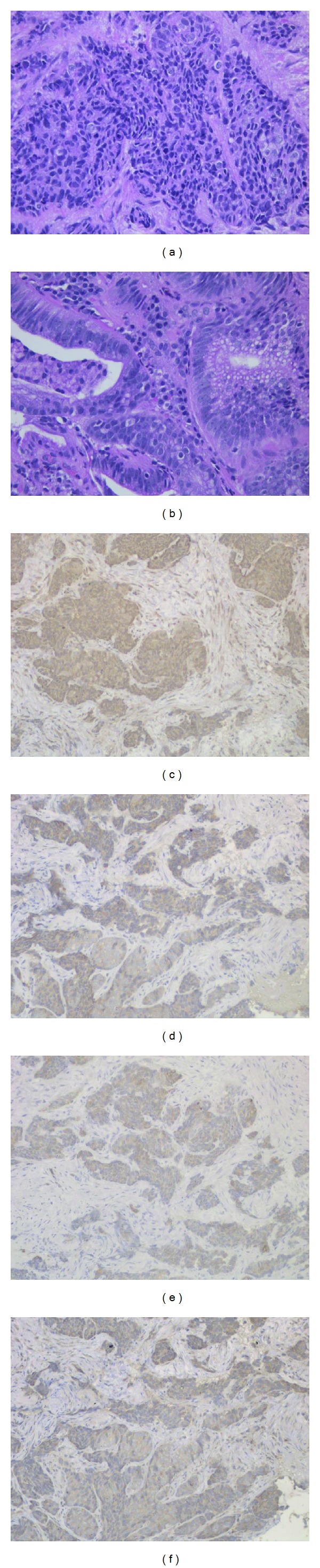
Proliferation of small and heterogeneous cancer cells with rich chromatin and fibrous septum with rich vessels at connective tissues was evident (hematoxylin and eosin) (a). Atypical cells forming glandular structures were evident (hematoxylin and eosin) (b). Immunostaining of small and heterogeneous cancer cells by chromogranin (c), synaptophysin (d), cytokeratin 7 (e), and EMA (f) was positive.

**Figure 3:**
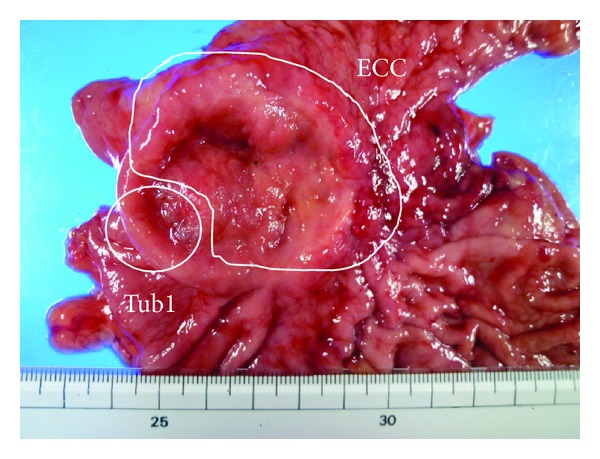
Distal gastrectomy with D2 lymph node dissection and Billroth I reconstruction was performed. Pathological examination revealed that adenocarcinoma cells comprised <10% of all cancer cells.

**Figure 4:**
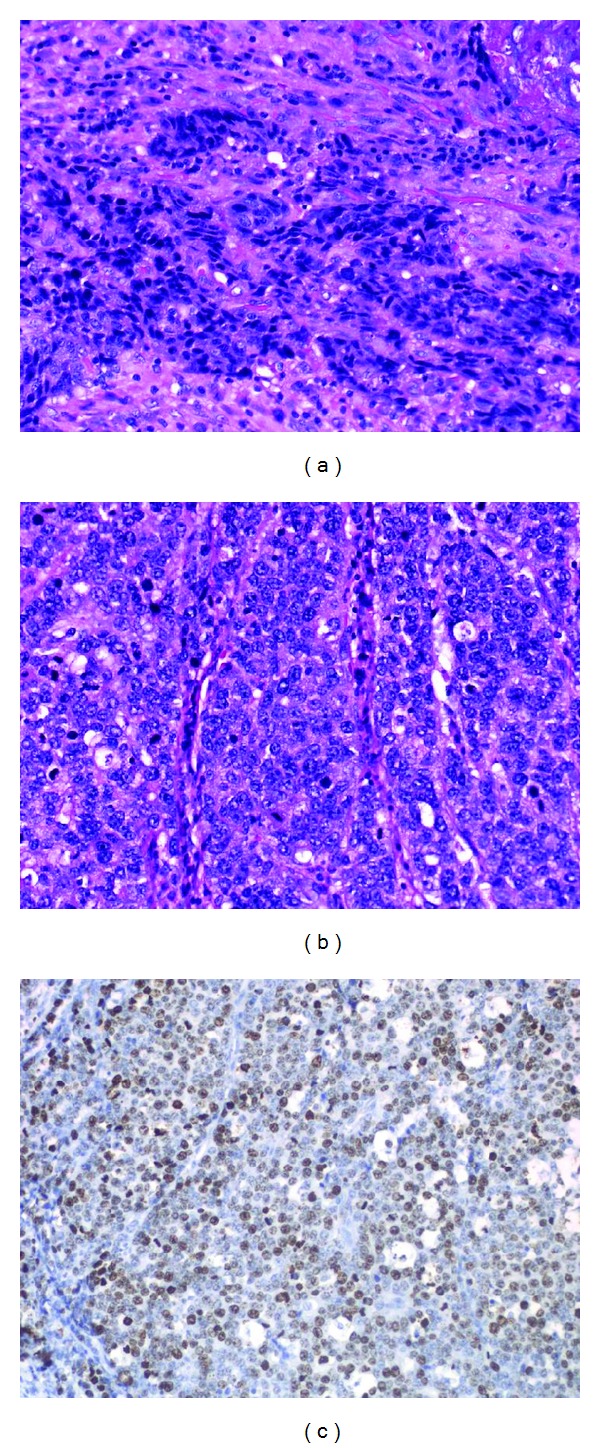
Microscopic examination of the resected specimen revealed the mixture of small (a) and large cells (b). Immunostaining by Ki-67 antibody revealed 58% of positive cells (c).

A 69-year-old female presented to our institution with epigastralgia and abdominal distension. Her past medical history included hypertension and dyslipidemia. On physical examination, her cognitive consciousness was alert. Her height, weight, and body mass index were 144.2 cm, 53.3 kg, and 25.6 kg/m^2^, respectively. Mild anemia was evident in her palpebral conjunctiva. Chest auscultation revealed no abnormal findings. The abdomen was soft and flat with normal bowel sounds. No lymph nodes were palpable, and no tumors were palpable on rectal examination. Blood chemistry analyses (Table 1) revealed mild anemia (red blood cell count, 347 × 10^4^/*μ*L; hemoglobin levels, 9.9 mg/dL), mildly increased C-reactive protein levels (0.6 mg/dL), mildly increased brain natriuretic peptide levels (55.6 pg/mL), and evidence of coagulation dysfunction (prothrombin time, 79%; international normalized ratio, 1.19). The tumor markers serum carcinoembryonic antigen (0.8 ng/mL) and serum carbohydrate antigen 19–9 (3.4 U/mL) were within normal limits. Chest radiography revealed cardiothoracic ratio of 51.4% without any evidence of pulmonary congestion or pleural effusion. Abdominal radiography revealed no abnormal gas distribution. Upper gastrointestinal series (Figure 1(a)) revealed a 5 cm ulcerative lesion with irregular margins and elevated distinct borders from the angle to the pyloric ring. Gastroendoscopy (Figure 1(b)) revealed a Borrmann type 2 tumor, extending from the angle to the antrum of the lesser curvature. The tumor comprised two different parts: ulcers with severe invasiveness and smooth protrusion from the endocrine cells (Figure 2(a)) and well-differentiated adenocarcinoma cells (Figure 2(b)) as exhibited by specimens biopsied under gastroendoscopy. ECC was confirmed by immunostaining examinations of cells, exhibiting positive results of chromogranin, synaptophysin, cytokeratin (CK) 7, and EMA (Figures 2(c)–2(f)). Other specimens revealed atypical cells forming glandular structures, leading to the diagnosis of well-differentiated tubular adenocarcinoma (tub1) (Figure 2(b)). Preoperative enhanced whole-body computed tomography (CT) revealed no distant metastases, signs of peritoneal dissemination, and regional lymph nodes swelling. Therefore, our gastric tumor was diagnosed as stage IIA tumor preoperatively. Subsequently, distal gastrectomy with D2 lymph node dissection and Billroth I reconstruction was performed. Pathological examination of the gross specimen (Figure 3(a)) revealed that adenocarcinoma cells comprised <10% of all cancer cells. Close analysis of ECC revealed a mixture of small and large cells, and 58% of Ki-67 labeling index (Figures 4(a)–4(c)). According to the WHO 2010 classification of gastrointestinal neuroendocrine tumors (NETs), ECC that coexists with adenocarcinoma (>30%) should be classified as “mixed adenoneuroendocrine carcinoma”. However, adenocarcinoma comprised <10% of all cancer cells; therefore, this gastric tumor was classified as NEC.

**Figure 5:**
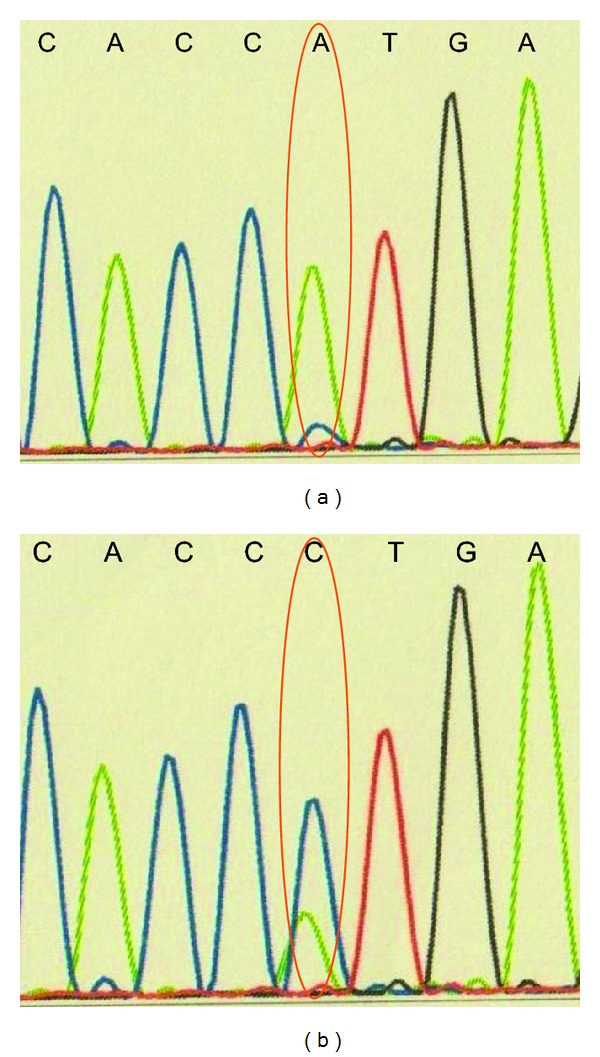
p53 gene sequences in exon 5–8 were analyzed. No alteration was identified in adenocarcinoma cells (a); in contrast, a transitional mutation (A to C) was identified in codon 179 of exon 5 in endocrine cells (b).

Postoperatively, this gastric cancer was diagnosed as M, Type 2, 50 × 50 mm, NEC, pT4a, INFa, ly(+), v(−), pN1 (4/38) of Stage IIIB. Immunostaining of endocrine and adenocarcinoma cells with p53 antibody revealed positive results for both components. Analysis indicated that transformation of pre-existing adenocarcinoma cells into endocrine cells is influenced by p53 gene alteration. In the present case, p53 gene sequences of exon 5–8, wherein most p53 gene mutations have been reported to occur, were analyzed, and transitional mutation (A to C) was identified in codon 179 of exon 5 in ECC (Figure 5). Although two types of cancers could develop separately, there might be the possibility that endocrine cells transformed from adenocarcinoma cells by p53 gene alteration.

Postoperative course was uneventful, and the patient was administered adjuvant chemotherapy with cisplatin and etoposide. One year following surgery, follow-up abdominal CT revealed multiple liver metastases. The patient received the best supportive care but eventually died 18 months after surgery.

## 3. Discussion

ECC composes highly atypical neoplastic endocrine cells and is a poor highly malignant cancer with rapid progression, early vessel invasion, early metastasis, and poor prognosis [2]. ECC is opposite of carcinoid, which is low atypical and low malignant. The clinical presentation of ECC is similar to that of adenocarcinoma except that it has a more biologically aggressive character. The mean survival time of ECC is 7 months [2]. Morphologically, most ECCs develop into Borrmann type 2 tumors; however, few predominantly develop into submucosal layers similar to carcinoid tumors and form ulcerated lesions along with severe invasion into submucosal layers, thereby exhibiting Borrmann type 3 tumors. ECC is usually treated using a combination of surgery and chemotherapy. Because the response rate of chemotherapy widely varies, no established regimen exists to date. ECC is treated using the treatment regimen for small cell lung carcinoma with cisplatin and etoposide, irinotecan, or paclitaxel [3, 4]. The gastric cancer regimen, S-1 with cisplatin, is also reportedly effective [5].

ECC comprises either small or large or both types of cells. In addition, the prevalence of coexistence of ECC with adenocarcinoma can be as high as 72.7% [1]. ECC is considered to develop as follows: differentiated glandular cancer cells arise initially, followed by the emergence of neoplastic endocrine cells (with high proliferative ability) through the differentiation of glandular cancer cells, and subsequently, endocrine cancer cells rapidly proliferate from the deep gland to submucosal layers, thereby forming glandular endocrine cancer.

Gastric ECC usually arises under submucosal layers, coexisting with mucosal adenocarcinoma. Normal mucosa covers over ECC; therefore, ECC is usually diagnosed as differentiated adenocarcinoma by specimens biopsied under endoscopy. With regard to the growth of ECC, the following four types of pathways have been hypothesized: (i) ECCs arise from pre-existing adenocarcinomas; (ii) ECCs arise from pre-existing carcinoid tumors: (iii) ECCs arise from nonneoplastic multipotent stem cells; (iv) ECCs arise from nonneoplastic endocrine cells. Of these, most gastrointestinal ECCs are considered to follow the first pathway [6].

## 4. Conclusions

When biopsied specimens reveal proliferation of small nuclear cancer cells without glandular structure, immunostaining should be performed to confirm ECC. Moreover, ECC occasionally comprised various types of cancer cells; therefore, endoscopic morphology of gastric ECC may vary according to the proportion of endocrine cancer cells. In the present case, during adjuvant chemotherapy following R0 resection, multiple liver metastases developed. Thus, future studies must focus on the emergence of promising anticancer agents or chemotherapy protocols.

## Figures and Tables

**Table 1:** Laboratory data on admission.

Hematology	
WBC	5,700 /*μ*L
RBC	347 × 10^4^ /*μ*L
Hb	9.9 g/dL
Ht	30.0%
MCV	86.5 fL
MCH	28.6 pg
MCHC	33.1 g/dL
PLT	37.0 × 10^4^ /*μ*L
Serology	
CRP	0.6 mg/dL
Blood chemistry	
TP	6.8 g/dL
Alb	4.0 g/dL
T-Bil	0.4 mg/dL
*γ*-GTP	16 IU/L
ALP	378 IU/L
AST	13 IU/L
ALT	14 IU/L
LDH	186 IU/L
CK	71 IU/L
BUN	9.9 mg/dL
Cr	0.60 mg/dL
Na	139 mEq/L
K	4.0 mEq/L
Cl	104 mEq/L
BNP	55.6 pg/mL
Coagulation	
PT	79%
PT-INR	1.19
APTT	29.7 sec
Sugar	
Glucose	125 mg/dL
Tumor marker	
CEA	0.8 ng/mL
CA19-9	3.4 U/mL
